# Supplementary material for: Methylene tetrahydrofolate reductase A1298C polymorphisms influence the adult sequelae of chemotherapy in childhood-leukemia survivors
Source: PLoS One. 2021 Apr 30;16(4):e0250228. doi: 10.1371/journal.pone.0250228 (PMC8087097; doi:10.1371/journal.pone.0250228)
Supplement: Attachment — Submitted filename: Rebuttal Letter.docx [file pone.0250228.s002.docx]

Leuven, September 26, 2020

Dear Editor,

Thank you for your essentially positive response to our manuscript. We addressed all reviewer comments below in our point-by-point reply. We appreciated the opportunity to improve our manuscript.

We hope the revised manuscript is now eligible for publication in *PLoS One*.

We apologize for the delay in revising the manuscript. As explicated before has the COVID-19 crisis hindered the availability of the first author, currently working as a medical doctor.

On behalf of all authors,

Iris Elens

Rudi D’Hooge

*Biological Psychology, KU Leuven, Belgium*

**We identified the following major comments:**

$$[\ldots]$$

(1) I have some concern regarding the strong conclusions made by the authors given the very small number of patients in two of the allele groups.

**Authors’ reply:**

Also see comment (3) and (6). We deleted the concluding paragraph and revised the discussion.

(2) In the methods the authors provide the number of participants for which Tau measurements were available at each of the treatment timepoints. However, there is no information on what allele group the missing participants were part of. It is therefore possible that the CC or AA groups could be even smaller for some of the analyses undertaken. This is of significant concern particularly for the CC group which only accounts for 5 participants. A breakdown of which group each of the ‘missing’ participants was part of for each of the timepoints is recommended to provide clarity regarding this issue.

**Authors’ reply:**

This information was added to the revised manuscript in table 1.

(3) The authors also frequently refer to data and descriptions that have been previously published. Although this is a widely accepted practice, it makes the data challenging to accurately place and interpret at times. Providing some minimal information on the sample, imaging approach etc. would be helpful to the reader.

**Authors’ reply:**

We are not entirely sure to which descriptions the reviewer refers, we provided more details of several of the cited studies. Also, we thoroughly revised the discussion, toned-down conclusion statements and referred more carefully to published results. As a result, almost every sentence of the discussion was rewritten or revised to address this comment.

(4) In regards to the results section, I am somewhat confused as to why information that is provided in the results is repeated in the figure legends. I would suggest that the figure legends simply identify what is represented by the figure, rather than repeating the analyses that were undertaken and the outcomes.

**Authors’ reply:**

On rereading, we found our figure legends indeed quite confusing and often redundant. Legends were completely rewritten and now provide a concise description of the figures.

(5) We are also not provided with any indication of how these participants perform in regards to their performance IQ and whether any participants exhibit clinically meaningful deficits in this area. This makes the meaningfulness of the relationship described between IQ and the alleles difficult to make sense of.

**Authors’ reply:**

This information was added to the revised manuscript, in the ‘results’ section.

(6) While I appreciate the approach taken by the authors to investigate this interesting question, the statements made in the conclusion are considered strong given the sample size and range of outcome measures. This is essentially a correlational study, however the wording in the conclusion suggests a causal relationship has been tested. This is somewhat misleading, particularly given that some of the possible relationships may not have been identified for the less common alleles because of a lack of power. Furthermore, some of the relationships identified are weak (e.g. correlation between PIQ and allele), but this has not been explicitly discussed in regards to the implications for the findings. The correspondence between these findings and those of Kamdar etc. who investigated these same alleles is also not covered in any detail in the conclusion, despite the fact that several of the findings are disparate.

**Authors’ reply:**

We agree with the reviewer that our observations should be treated with caution and might even be considered to be preliminary in many respects. We revised the discussion extensively and toned down all our statements that could be perceived as “too bold” for such a correlational study. We also deleted the last sentence of the abstract and revised the penultimate sentence. We do not understand why this reviewer writes that the findings of Kamdar et al. are disparate from ours. In fact, Kamdar et al reported, for instance, that “survivors with MTHFR 1298AC/CC genotypes scored, on average, 13 points lower on TMTB than those with MTHFR 1298AA genotype” (*Pediatr Blood Cancer* **57**: 454–460, 2011).

**We identified the following minor comments:**

(1) In the hypotheses the authors refer to “read-outs”. It is unclear what this means, and whether they are referring to outcomes.
(2) In Table 1:
    a. Abbreviations of VLR, AR1 and AR2 are used but not explained. There should be reference to these here even though they are described in the following table.
    b. SES – the authors make reference to ‘as described earlier’ but there is no description prior to this in the document. If the authors are referring to their previous publication then this should be clearly indicated here, with the reference provided.
(3) In Table 2: *** are used to refer to methotrexate and cyclophosphamide, but only MTX + leucovorin rescue is highlighted in the footnotes.
(4) Figure 1 – error in line 5, ‘vor’
(5) Figure 2 description line 3 – but slightly not Tau?
(6) Figure 4 – Line 2, missing M in THFR
(7) In discussion – when discussing ODI, the comment is made (but slightly not ODI)
(8) Sentence ‘Particular white matter sensitivity…..’ is not immediately interpretable. Requires further explanation
(9) in regards to allowing access to the data, while the authors have responded with 'yes' to this question, it is then stated that the data is only available on request. This requires amendment.
(10) Cecilia Papp wrote for PLOS ONE: "From all participants (including parents in case of minors), written informed consent was obtained." could you please also add the consent statement to your ethics statement in the methods section of your manuscript.

This information had been added to the manuscript.

**All these comments have been addressed.**
