# Supplementary material for: Methylene tetrahydrofolate reductase A1298C polymorphisms influence the adult sequelae of chemotherapy in childhood-leukemia survivors
Source: PLoS One. 2021 Apr 30;16(4):e0250228. doi: 10.1371/journal.pone.0250228 (PMC8087097; doi:10.1371/journal.pone.0250228)
Supplement: Attachment — Submitted filename: Manuscript Rebuttal R3.docx [file pone.0250228.s003.docx]

**Rebuttal letter – revision R3**

Manuscript PONE-D-19-35942R1: “Methylene tetrahydrofolate reductase A1298C polymorphisms influence the adult sequelae of chemotherapy in childhood-leukemia survivors” by Elens et al.

*Rebuttal letter by Iris Elens and Rudi D’Hooge, on behalf of all co-authors*

*University of Leuven, Belgium*

**We revised our manuscript according to the following “Additional Editor Comments”:**

*Some minor errors detected" their MTHFR1298 genotype influenced changes in PIQ". It is unclear that which MTHFR genotype are they referring to. It is unclear that which MTHFR genotype are they referring to. It is also unclear what is missing data Tau 2, 7, ....listed in the table. It is also unclear what is missing data Tau 2, 7, ....as listed in the table. This sentence is unclear "They were made aware that they could withdraw at any time from the study, without justification as to why they did so. ". Typo "PIQ was 93.800, 93.411 and 106.714 for CC, AC and AA genotype, respectively' & 'We report that individuals with the MTHFR1209CC genotype presented the highest FA at the left centrum semiovale' & 'This is in line with previous observations. Winick et al.33 reported ........."*

**Not all of these comments were clear to us, but we accept that some of these parts were not written as clearly as possible.**

Changes in the revised manuscript R3:

(1) *It is also unclear what is missing data Tau 2, 7, ....listed in the table. It is also unclear what is missing data Tau 2, 7, ....as listed in the table.*

**We accept that this was not very clear. We therefore removed these numbers of missing data from Table 1, and included a new Table 2 that lists the different missing data from the incomplete patient dossiers. We also rewrote the corresponding text in the methods section as well.**

(2) *This sentence is unclear "They were made aware that they could withdraw at any time from the study, without justification as to why they did so.”*

**We were not certain what was meant here, but we rewrote this sentence and included additional information about the ICF and the IC procedures at the UZ Leuven university hospital.**

(3) *Typo "PIQ was 93.800, 93.411 and 106.714 for CC, AC and AA genotype, respectively' & 'We report that individuals with the MTHFR1209CC genotype presented the highest FA at the left centrum semiovale' & 'This is in line with previous observations. Winick et al.33 reported ........."*

**We rewrote these sentences.**
